# In Memoriam—David Wilson, M. D.

**Published:** 1889-12

**Authors:** 


### IN MEMORIAM—DAVID WILSON, M. D.

Another of Homœopathy's greatest practitioners has been taken from us! Indeed, in announcing the death of David Wilson, we tell of the fall of one of the ablest and most successful disciples of Hahnemann. Dr. Wilson ranked with Hering, Lippe, and Bayard; none ever followed more carefully the teachings of Hahnemann, and few, if any, ever achieved greater success in curing the sick than did Dr. Wilson.

David Wilson was born at Duns, a small village near Berwick-on-Tweed, Scotland, in 1811. Having received his school education in his native village and at the High School of Edinburgh, he entered as a student of the Extra-Academical Medical School connected with the Edinburgh College of Surgeons, of which body he became a licentiate in 1829. Like many others of his countrymen, he came south, hoping to find an El Dorado in London, and actually walked from Edinburgh to London, starting with £20 in his pocket. In this he was disappointed, and consequently accepted a berth as surgeon to a vessel bound for the East Coast of Africa and the Seychelles Islands. In this way he passed three years, and on his return to London he procured a situation as assistant to the late Dr. Hastings, of Eccleston Square, with whom, in a few years, he entered into partnership, and in conjunction with whom he carried on a very extensive and lucrative practice. It was during this time that, lamenting the inefficacious and oftentimes injurious methods of practice then universally adopted, and ever on the alert to hear of something better, Homœopathy was introduced to his notice, and he diligently studied such books as could be obtained upon the subject, and more especially did he strive to master *The Organon* of Hahnemann. That he might do so the more perfectly, he learned German. During this period he also made many experiments of a clinical character, prescribing homœopathically for cases that had baffled the skill both of himself and his partner. The results were so striking and so satisfactory that in 1849 he dissolved partnership with Dr. Hastings, and devoted himself to the practice and dissemination of a knowledge of Homœopathy. About this period he established *The Homœopathic Times*—a weekly journal—which battled valiantly for the truth for a few years.

Dr. Wilson's strong convictions and intense earnestness for the cause made him bold and outspoken in its defense. He never hesitated in his determined exposure of mongrelism on the one hand, nor, on the other, to avow his conviction as to the truth of Hahnemann's teachings. Dr. Wilson was one of the founders of the Hahnemann Hospital of London, and for a time he assisted in its management. Finally, seeing that his colleagues in the hospital were not strict homœopathists, he withdrew.

For thirty-nine years, Dr. Wilson lived and practiced in Brook Street, London ; it is needless to add that his practice was extensive and most successful.

Dr. Wilson was a man who had the faculty of inspiring the most thorough confidence in his patients, all of whom were much attached to him. His great earnestness, the deep sense he entertained of the truth of Homœopathy, and the readiness with which he went through any amount of trouble to relieve his patients and to propagate a knowledge of Homœopathy, have enabled him to be the means of doing a large amount of useful work during the last forty years. A thorough, staunch Hahnemannian, he never swerved for one moment from the strict rules of practice laid down by that great man, Samuel Hahnemann, and was entirely opposed to any other means of cure. The only exception, if it can be called such, was his adoption of the use of Massage, anatomically and scientifically applied, in cases he thought would be materially aided by it. In 1864 the Homœopathic Medical College of Pennsylvania conferred upon him its honorary degree of M. D. "for zeal to the cause, high attainments, and the excellence of literary and scientific labors." He was ever ready to stretch out his helping hand to any student desirous of learning Homœopathy, and several used to attend his private dispensary and learn the clinical lessons in Homœopathy inculcated by him. He died suddenly of syncope, brought on by over-work and mental strain. It may be truly said of him that he died "in harness."

